# Metabolic engineering of *Escherichia coli* for efficient production of d-phenylglycine

**DOI:** 10.1016/j.synbio.2026.07.010

**Published:** 2026-08-07

**Authors:** Yajuan Xiong, Zhentong Shang, Liangyu Lu, Tong Wang, Guang Cai, Xiaolin Shen, Xinxiao Sun, Jia Wang, Qipeng Yuan

**Affiliations:** a College of Life Science and Technology, Beijing University of Chemical Technology, Beijing, 100029, China; b Beijing Advanced Innovation Center for Soft Matter Science and Engineering, Beijing University of Chemical Technology, Beijing, 100029, China; c State Key Laboratory of Green Biomanufacturing, Beijing, China

**Keywords:** d-phenylglycine, Biosynthesis, Metabolic engineering, *Escherichia coli*

## Abstract

d-phenylglycine (D-PHG) is a valuable building block extensively used in the synthesis of β-lactam antibiotics and other high-value pharmaceuticals. However, its conventional chemical production relies on expensive starting materials and harsh reaction conditions, while existing biotechnological approaches remain constrained by low production efficiency. In the present work, we established a novel *de novo* biosynthetic pathway for D-PHG production from glucose in *Escherichia coli* by reconstructing a streamlined route from the native shikimate pathway intermediate phenylpyruvate. The pathway integrates 4-hydroxymandelate synthase, an FMN-dependent *S*-mandelate dehydrogenase that avoids H_2_O_2_ formation, and a highly specific d-phenylglycine aminotransferase, enabling efficient D-PHG production. Systematic metabolic engineering strategies, including elimination of competing pathways, enhancement of precursor supply, optimization of amino group donor availability, and reinforcement of NADPH regeneration, increased the D-PHG titer to 6.98 g/L in shake-flask cultivation. Further scale-up in a 3-L fed-batch fermentation achieved 9.83 g/L, representing the highest reported titer for *de novo* D-PHG biosynthesis from glucose to date. This work establishes an efficient and sustainable biosynthetic route to D-PHG and provides a foundation for its large-scale industrial manufacturing.

## Graphical abstract

**Figure:**
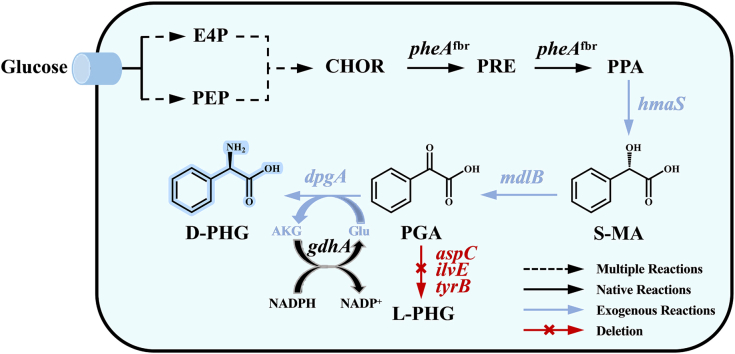

## 1 Introduction

d-Phenylglycine (D-PHG), a noncanonical chiral amino acid, serves as a key intermediate in the manufacture of β-lactam antibiotics including ampicillin, cephalexin, cephradine, cefaclor, and penicillin G [[1], [2], [3], [4]]. In addition, D-PHG functions as a key building block for the preparation of SA16, a dual inhibitor of phosphoinositide-dependent kinase-1 (PDK1) and Aurora kinase A (AurA), which modulates kinase activity and induces tumor cell differentiation and apoptosis [5,6]. D-PHG can also be used as an intermediate in the synthesis of agrochemicals such as chlorfenapyr [7]. In 2024, the global market for D-PHG was valued at approximately USD 87.46 million and is expected to increase to USD 126 million by 2031 [8].

Traditionally, D-PHG has been produced mainly through chemical synthesis, with the Bucherer-Bergs route [1,9] and the glyoxylic acid route [10] being the most widely employed. However, both approaches suffer from significant inherent limitations. In the Bucherer-Bergs route, benzaldehyde is converted into DL-PHG through cyclization in the presence of cyanide and ammonia or ammonium carbonate, followed by hydrolysis and acidification under harsh conditions of high temperature and pressure. This process depends on highly toxic cyanide reagents and stringent reaction conditions, raising significant environmental and safety concerns. The glyoxylic acid route utilizes glyoxylic acid, benzene, and acetamide to synthesize N-acetylphenylglycine, which is subsequently deacetylated to yield DL-PHG. However, glyoxylic acid is highly reactive and prone to self-condensation and side reactions, such as over-acetylation, leading to complex by-product profiles and increased downstream purification costs. Notably, both methods produce racemic mixtures, necessitating an additional, costly, and technically demanding chiral resolution step to obtain optically pure D-PHG, thereby limiting their industrial applicability.

Recently, several studies have explored the biotechnological production of D-PHG. One representative approach employs racemic mandelate as an exogenous substrate in a multi-enzyme cascade. In this system, mandelate racemase from *Pseudomonas putida* dynamically racemizes mandelate, while S-mandelate dehydrogenase selectively oxidizes S-mandelate (S-MA) to phenylglyoxylate (PGA). d-phenylglycine aminotransferase (D-PhgAT) from *P. stutzeri* ST-201 then converts PGA into D-PHG with l-glutamate serving as the amino donor, with glutamate dehydrogenase from *E. coli* enabling cofactor recycling. This system achieves efficient conversion, producing 29.5 g/L D-PHG from 210 mM mandelate with a 93% yield [11]. However, this strategy relies on costly racemic mandelate and requires stepwise substrate feeding and sequential biocatalytic reactions, resulting in operational complexity that limits its suitability for continuous industrial processes. To address these limitations, *de novo* biosynthesis of D-PHG from glucose has been investigated. In this pathway, 4-hydroxymandelate synthase HmaS from *Amycolatopsis orientalis* catalyzes the transformation of phenylpyruvate (PPA) into S-MA, which is subsequently oxidized to PGA by 4-hydroxymandelate oxidase Hmo from *Streptomyces coelicolor*. PGA is then transaminated to D-PHG by 4-hydroxyphenylglycine aminotransferase HpgAT from *P. putida*. Despite its conceptual advantages, this route achieves only 102 mg/g CDW [12], far below industrially relevant levels. This limitation is primarily attributed to insufficient precursor supply. In addition, Hmo uses molecular oxygen as the electron acceptor and generates hydrogen peroxide (H_2_O_2_) as a by-product, which can inactivate enzymes and impose oxidative stress on the host, thereby compromising cell growth and productivity.

**Fig. 1:**
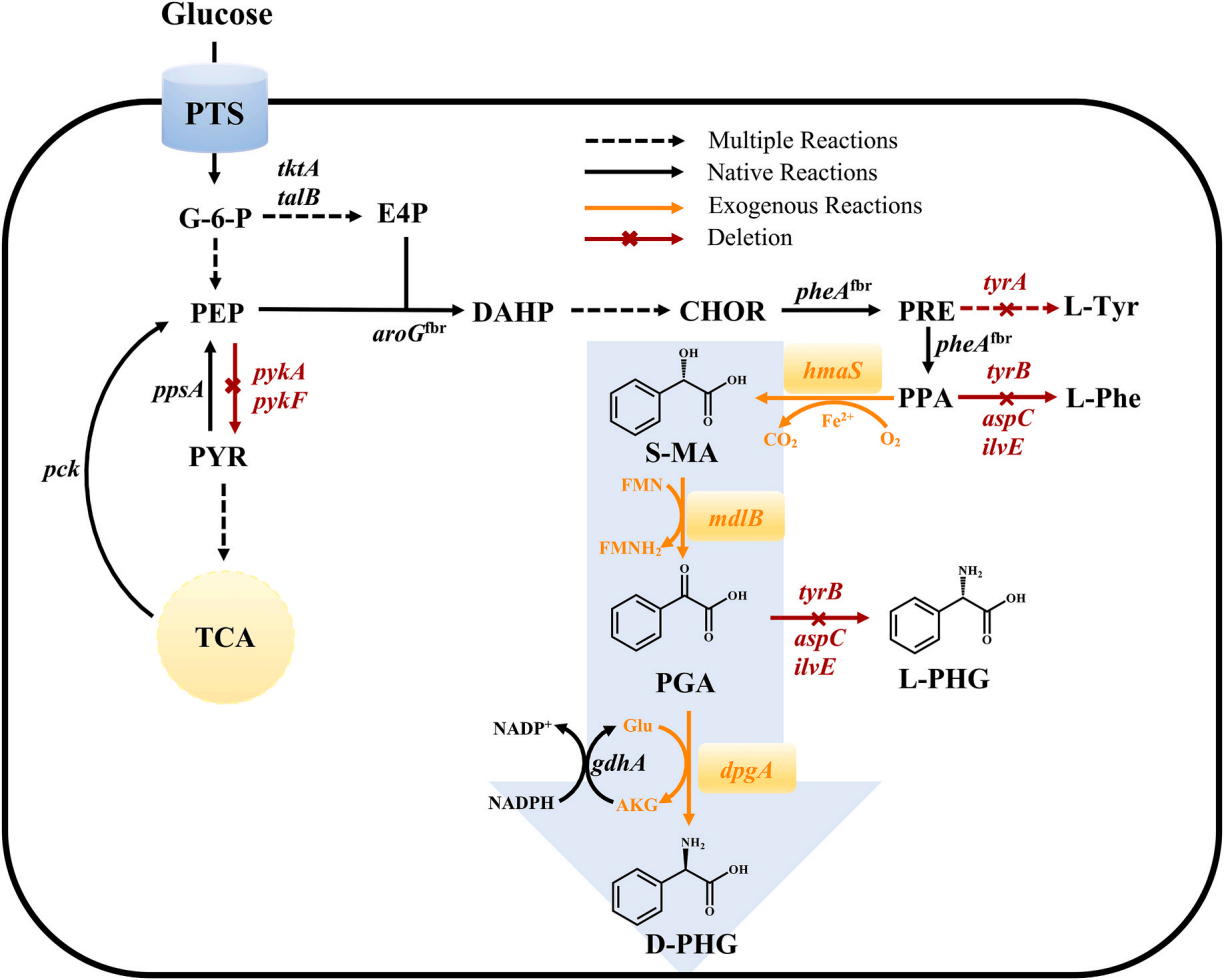
*De novo* biosynthetic pathway for D-PHG production. TCA: tricarboxylic acid cycle; PTS: PEP-dependent phosphotransferase system; G-6-P: glucose-6-phosphate; E4P: erythrose-4-phosphate; PEP: phosphoenolpyruvate; PYR: pyruvate; DAHP: 3-deoxy-D-arabino-heptulosonate-7-phosphate; CHOR: chorismate; PRE: prephenate; PPA: phenylpyruvate; L-Tyr: l-tyrosine; L-Phe: l-phenylalanine; S-MA: S-mandelate; PGA: phenylglyoxylate; D-PHG: d-phenylglycine; L-PHG: l-phenylglycine; O_2_: oxygen; CO_2_: carbon dioxide; Fe^2+^: ferrous ion; FMN: flavin mononucleotide; FMNH_2_: reduced flavin mononucleotide; NADP^+^: nicotinamide adenine dinucleotide phosphate; NADPH: reduced nicotinamide adenine dinucleotide phosphate; genes: *pykA*/*F*, encoding pyruvate kinase; *aroG*^fbr^, encoding the feedback-resistant mutant of 3-deoxy-7-phosphoheptulonate synthase; *pheA*^fbr^, encoding the feedback-resistant mutant of prephenate dehydratase; *tyrA*, encoding prephenate dehydrogenase; *tyrB*, encoding aromatic amino acid aminotransferase; *aspC*, encoding aspartate aminotransferase; *ilvE*, encoding branched-chain amino acid aminotransferase; *hmaS*, encoding 4-hydroxymandelate synthase; *mdlB*, encoding S-mandelate dehydrogenase; *dpgA*, encoding d-phenylglycine aminotransferase; *gdhA*, encoding glutamate dehydrogenase. Orange solid arrows represent exogenous reactions; black solid arrows represent native reactions; red arrows with red X marks represent gene deletions; black dashed arrows represent multiple reactions.

In this study, we reconstituted and optimized a biosynthetic route for D-PHG to enable its efficient production from glucose (Fig. 1). First, endogenous pathways leading to the by-product l-phenylglycine were disrupted to eliminate competing flux and ensure product enantiopurity. Then, HmaS from *A. orientalis* was introduced to catalyze the conversion of PPA to S-MA. S-mandelate dehydrogenase SMDH from *P. putida*, an FMN-dependent dehydrogenase, was employed to convert S-MA into PGA, thereby avoiding H_2_O_2_ formation. Subsequently, D-PhgAT from *P. stutzeri* ST-201, exhibiting high catalytic specificity, was used to convert PGA to D-PHG, resulting in an initial titer of 2.79 g/L. To further improve production, we redirected more carbon flux into the shikimate route and strengthened the intracellular supply of phosphoenolpyruvate (PEP) and erythrose-4-phosphate (E4P), increasing the D-PHG titer to 4.94 g/L. Additional metabolic engineering strategies, including tuning D-PhgAT expression level, reinforcing amino group donor availability, and optimizing NADPH regeneration, further boosted the titer to 6.98 g/L in shake-flask cultivation. Finally, scale-up in a 3-L fed-batch fermentation process achieved a D-PHG titer of 9.83 g/L, representing the highest reported titer for *de novo* D-PHG biosynthesis from glucose to date. Overall, this study establishes a microbial platform for D-PHG biosynthesis, paving the way for its cost-effective and sustainable industrial-scale manufacturing.

## 2 Materials and methods

### 2.1 Strains and plasmids

Plasmids were constructed using the strain *E. coli* Trans5α. *E. coli* BW25113 was used as the host strain for target product biosynthesis. The biosynthetic pathway was assembled using the plasmids pET-lac (high-copy-number), pCS27 (medium-copy-number), and pSA74 (low-copy-number) [13,14]. Plasmids pTarget and pCas9 were used for CRISPR/Cas9-mediated genome editing. Detailed information on the strains and plasmids employed in this study is provided in Table 1, Table 2.

**Table 1:** Strains used in this study.

Strain	Genotype	Source
*E. coli* BW25113	*rrnBT14* △*lacZWJ16 hsdR514*△*araBADAH33*△*rhaBADLD78*	Yale CGSC
*E. coli* Trans5α	*F-φ80d lacZ*Δ*M*15 Δ*(lacZYA-argF) U169 end A1 recA1 hsdR17 (rk*−*, mk*^+^*) supE44λ- thi-1 gyrA96 relA1 phoA*	Stratagene
XYJ01	BW25113 Δ*tyrB*, Δ*aspC*, Δ*ilvE*	This study
XYJ02	BW25113 Δ*pykA*, Δ*pykF*, Δ*tyrA*, Δ*tyrB*,Δ*aspC*, Δ*ilvE*, Δ*tyrR*, Δ*ilvG*::P_lpp4.2_*-aroG*^fbr^*-aroB-aroD*	This study
XYJ03	XYJ02 Δ*yeeL*::P_50trc_-*gdhA*	This study
XYJ04	XYJ03 Δ*yciQ*::P_50trc_-*nadK*	This study
XYJ05	XYJ04 Δ*ylbE*::P_10trc_-*pntAB*	This study
XYJ06	BW25113 carrying pET-lac	This study
XYJ07	XYJ01 carrying pET-lac	This study
XYJ08	XYJ02 carrying pET-lac-*hmaS*-*hmaS*-*mdlB*-lac-*hmaS* and pCS-P_lpp0.6_-*dpgA*	This study
XYJ09	XYJ02 carrying pET-lac-*hmaS*-*hmaS*-*mdlB*-lac-*hmaS* and pCS-P_lpp0.6_-*dpgA*-lac-*aroG*^fbr^	This study
XYJ10	XYJ02 carrying pET-lac-*hmaS*-*hmaS*-*mdlB*-lac-*hmaS* and pCS-P_lpp0.6_-*dpgA*-lac-*pheA*^fbr^	This study
XYJ11	XYJ02 carrying pET-lac-*hmaS*-*hmaS*-*mdlB*-lac-*hmaS* and pCS-P_lpp0.6_-*dpgA*-lac-*pheA*^fbr^-*aroG*^fbr^	This study
XYJ12	XYJ02 carrying pET-lac-*hmaS*-*hmaS*-*mdlB*-lac-*hmaS* and pCS-P_lpp0.6_-*dpgA*-lac-*pheA*^fbr^-*ppsA*	This study
XYJ13	XYJ02 carrying pET-lac-*hmaS*-*hmaS*-*mdlB*-lac-*hmaS* and pCS-P_lpp0.6_-*dpgA*-lac-*pheA*^fbr^-*pck*	This study
XYJ14	XYJ02 carrying pET-lac-*hmaS*-*hmaS*-*mdlB*-lac-*hmaS* and pCS-P_lpp0.6_-*dpgA*-lac-*pheA*^fbr^-*ppsA*-*pck*	This study
XYJ15	XYJ02 carrying pET-lac-*hmaS*-*hmaS*-*mdlB*-lac-*hmaS* and pCS-P_lpp0.6_-*dpgA*-lac-*pheA*^fbr^-*talB-tktA*	This study
XYJ16	XYJ02 carrying pET-lac-*hmaS*-*hmaS*-*mdlB*-lac-*hmaS* and pCS-P_lpp0.6_-*dpgA*-lac-*pheA*^fbr^-*ppsA*-*pck*-*talB*-*tktA*	This study
XYJ17	XYJ02 carrying pET-lac-*hmaS*-*hmaS*-*mdlB*-lac-*hmaS* and pCS-P_lpp0.2_-*dpgA*-lac-*pheA*^fbr^-*ppsA*-*pck*-*talB*-*tktA*	This study
XYJ18	XYJ02 carrying pET-lac-*hmaS*-*hmaS*-*mdlB*-lac-*hmaS* and pCS-lac-*dpgA*-lac-*pheA*^fbr^-*ppsA*-*pck*-*talB*-*tktA*	This study
XYJ19	XYJ02 carrying pET-lac-*hmaS*-*hmaS*-*mdlB*-lac-*hmaS* and pCS-P_lpp1.0_-*dpgA*-lac-*pheA*^fbr^-*ppsA*-*pck*-*talB*-*tktA*	This study
XYJ20	XYJ02 carrying pET-lac-*hmaS*-*hmaS*-*mdlB*-lac-*hmaS* and pCS-P_lpp4.0_-*dpgA*-lac-*pheA*^fbr^-*ppsA*-*pck*-*talB*-*tktA*	This study
XYJ21	XYJ03 carrying pET-lac-*hmaS*-*hmaS*-*mdlB*-lac-*hmaS* and pCS-lac-*dpgA*-lac-*pheA*^fbr^-*ppsA*-*pck*-*talB*-*tktA*	This study
XYJ22	XYJ03 carrying pET-lac-*hmaS*-*hmaS*-*mdlB*-lac-*hmaS*, pCS-lac-*dpgA*-lac-*pheA*^fbr^-*ppsA*-*pck*-*talB*-*tktA* and pSA-lac-*maeB*	This study
XYJ23	XYJ03 carrying pET-lac-*hmaS*-*hmaS*-*mdlB*-lac-*hmaS*, pCS-lac-*dpgA*-lac-*pheA*^fbr^-*ppsA*-*pck*-*talB*-*tktA* and pSA-lac-*zwf*	This study
XYJ24	XYJ03 carrying pET-lac-*hmaS*-*hmaS*-*mdlB*-lac-*hmaS* pCS-lac-*dpgA*-lac-*pheA*^fbr^-*ppsA*-*pck*-*talB*-*tktA* and pSA-lac-*pntAB*	This study
XYJ25	XYJ03 carrying pET-lac-*hmaS*-*hmaS*-*mdlB*-lac-*hmaS*, pCS-lac-*dpgA*-lac-*pheA*^fbr^-*ppsA*-*pck*-*talB*-*tktA* and pSA-lac-*nadK*	This study
XYJ26	XYJ03 carrying pET-lac-*hmaS*-*hmaS*-*mdlB*-lac-*hmaS*, pCS-lac-*dpgA*-lac-*pheA*^fbr^-*ppsA*-*pck*-*talB*-*tktA* and pSA-lac-*ppnK*	This study
XYJ27	XYJ04 carrying pET-lac-*hmaS*-*hmaS*-*mdlB*-lac-*hmaS* and pCS-lac-*dpgA*-lac-*pheA*^fbr^-*ppsA*-*pck*-*talB*-*tktA*	This study
XYJ28	XYJ05 carrying pET-lac-*hmaS*-*hmaS*-*mdlB*-lac-*hmaS* and pCS-lac-*dpgA*-lac-*pheA*^fbr^-*ppsA*-*pck*-*talB*-*tktA*	This study

**Table 2:** Plasmids used in this study.

Plasmid	Description	Source
pET-lac	pLlacO1; *ColE ori*; *Amp*^R^	[13]
pCS27	pLlacO1; *p15A ori*; *Kan*^R^	[15]
pSA74	pLlacO1, *pSC101 ori*, *Cm*^R^	[15]
pET-lac-*hmaS*-*hmaS*-*mdlB*	pET-lac containing *hmaS* (AEI58881.1) from *A*. *orientalis and mdlB* (P20932.1) from *P*. *putida* ATCC 12633	This study
pET-lac*-hmaS*-*hmaS*-*mdlB*-lac-*hmaS*	pET-lac-*hmaS*-*hmaS*-*mdlB* containing *hmaS* (AEI58881.1) from *A*. *orientalis*	This study
pCS-P_lpp0.6_-*dpgA*	pCS27 containing *dpgA* from *P. stutzeri* ST-201 (Q6VY99) under the P_lpp0.6_ promoter	This study
pCS-P_lpp0.6_-*dpgA*-lac-*aroG*^fbr^	pCS-P_lpp0.6_-*dpgA* containing *aroG*^fbr^ (AXN70009.1) from *E*. *coli*	This study
pCS-P_lpp0.6_-*dpgA*-lac-*pheA*^fbr^	pCS-P_lpp0.6_-*dpgA* containing *pheA*^fbr^ (ACT66712.1) from *E*. *coli*	This study
pCS-P_lpp0.6_-*dpgA*-lac-*pheA*^fbr^*-aroG*^fbr^	pCS-P_lpp0.6_-*dpgA*-lac-*pheA*^fbr^ containing *aroG*^fbr^ (AXN70009.1) from *E. coli*	This study
pCS-P_lpp0.6_-*dpgA*-lac-*pheA*^fbr^*-ppsA*	pCS-P_lpp0.6_-*dpgA*-lac-*pheA*^fbr^ containing *ppsA* (P23538) from *E. coli*	This study
pCS-P_lpp0.6_-*dpgA*-lac-*pheA*^fbr^*-pck*	pCS-P_lpp0.6_-*dpgA*-lac-*pheA*^fbr^ containing *pck* (P22259) from *E. coli*	This study
pCS-P_lpp0.6_-*dpgA*-lac-*pheA*^fbr^*-ppsA-pck*	pCS-P_lpp0.6_-*dpgA*-lac-*pheA*^fbr^-*ppsA* containing *pck* from *E. coli*	This study
pCS-P_lpp0.6_-*dpgA*-lac-*pheA*^fbr^*-talB-tktA*	pCS-P_lpp0.6_-*dpgA*-lac-*pheA*^fbr^ containing *talB* (P0A870) and *tktA* (P27302) from *E. coli*	This study
pCS-P_lpp0.6_-*dpgA*-lac-*pheA*^fbr^*-ppsA-pck-talB-tktA*	pCS-P_lpp0.6_-*dpgA*-lac-*pheA*^fbr^*-ppsA-pck* containing *talB* and *tktA* from *E. coli*	This study
pCS-P_lpp0.2_-*dpgA*-lac-*pheA*^fbr^*-ppsA-pck-talB-tktA*	pCS-P_lpp0.6_-*dpgA*-lac-*pheA*^fbr^*-ppsA-pck-talB-tktA* under the P_lpp0.2_ promoter	This study
pCS-lac-*dpgA*-lac-*pheA*^fbr^*-ppsA-pck-talB-tktA*	pCS-P_lpp0.6_-*dpgA*-lac-*pheA*^fbr^*-ppsA-pck-talB-tktA* under the P_LlacO1_ promoter	This study
pCS-P_lpp1.0_-*dpgA*-lac-*pheA*^fbr^*-ppsA-pck-talB-tktA*	pCS-P_lpp0.6_-*dpgA*-lac-*pheA*^fbr^*-ppsA-pck-talB-tktA* under the P_lpp1.0_ promoter	This study
pCS-P_lpp4.0_-*dpgA*-lac-*pheA*^fbr^*-ppsA-pck-talB-tktA*	pCS-P_lpp0.6_-*dpgA*-lac-*pheA*^fbr^*-ppsA-pck-talB-tktA* under the P_lpp4.0_ promoter	This study
pSA-lac-*maeB*	pSA74 containing *maeB* (P76558) from *E. coli*	This study
pSA-lac-*zwf*	pSA74 containing *zwf* (P0AC53) from *E. coli*	This study
pSA-lac-*pntAB*	pSA74 containing *pntA* (P07001) and *pntB* (P0AB67) from *E. coli*	This study
pSA-lac-*nadK*	pSA74 containing *nadK* (P0A7B3) from *E. coli*	This study
pSA-lac-*ppnK*	pSA74 containing *ppnK* (Q8NQM1) from *C*. *glutamicum*	This study

### 2.2 Medium

Luria-Bertani (LB) medium was used for plasmid construction and strain cultivation. Shake-flask fermentation was performed in M9Y medium supplemented with 5 g/L yeast extract, 2 g/L MOPS, 1 g/L NH_4_Cl, 0.5 g/L NaCl, 3 g/L KH_2_PO_4_, 6.78 g/L Na_2_HPO_4_, 30 g/L glucose, 1 mM MgSO_4_, and 0.1 mM CaCl_2_. During cultivation, the appropriate antibiotics were supplemented as required, including kanamycin (50 μg/mL), ampicillin (100 μg/mL), and chloramphenicol (25 μg/mL). According to the requirements of different strains, the medium was additionally fortified with 3 g/L aspartate, 0.2 g/L tyrosine, and 0.1 g/L each of leucine, isoleucine, phenylalanine, and valine.

### 2.3 DNA Manipulation

The *hmaS* gene from *A. orientalis* was codon-optimized for expression in *E. coli*. The *mdlB* gene was amplified by PCR from the genomic DNA of *P. putida* ATCC 12633, and the codon-optimized *hmaS* together with *mdlB* was cloned into plasmid pET-lac using the restriction sites *Kpn*I, *Hin*dIII, *Xba*I, and *Bam*HI, yielding pET-lac-*hmaS*-*hmaS*-*mdlB*. The expression cassette P_LlacO1_-*hmaS* was inserted into the *AvrII* site of plasmid pET-lac-*hmaS*-*hmaS*-*mdlB* to generate pET-lac-*hmaS*-*hmaS*-*mdlB-*lac*-hmaS*. The *dpgA* gene was amplified from the genome of *P. stutzeri* ST-201 and cloned into pCS27 vector under the control of the P_lpp0.6_ promoter using the restriction sites *Kpn*I and *Bam*HI, yielding plasmid pCS-P_lpp0.6_-*dpgA*. The expression cassette P_LlacO1_-*pheA*^fbr^, P_LlacO1_-*aroG*^fbr^, and P_LlacO1_-*pheA*^fbr^-*aroG*^fbr^ were inserted between the *Bcu*I and *Sac*I sites of plasmid pCS-P_lpp0.6_-*dpgA* to construct plasmids pCS-P_lpp0.6_-*dpgA*-lac-*pheA*^fbr^, pCS-P_lpp0.6_-*dpgA*-lac-*aroG*^fbr^, and pCS-P_lpp0.6_-*dpgA*-lac-*pheA*^fbr^*-aroG*^fbr^, respectively. Subsequently, the *ppsA* and *pck* genes, together with the *talB*-*tktA* expression module, all derived from *E. coli* MG1655 were individually inserted into pCS-P_lpp0.6_-*dpgA*-lac-*pheA*^fbr^ by homologous recombination to construct plasmids pCS-P_lpp0.6_-*dpgA*-lac-*pheA*^fbr^-*ppsA*, pCS-P_lpp0.6_-*dpgA-*lac-*pheA*^fbr^-*pck*, and pCS-P_lpp0.6_-*dpgA*-lac-*pheA*^fbr^-*talB*-*tktA*. Likewise, the *pck* gene and the *talB*-*tktA* expression cassette were sequentially introduced into pCS-P_lpp0.6_-*dpgA*-lac-*pheA*^fbr^-*ppsA*, generating plasmids pCS-P_lpp0.6_-*dpgA*-lac-*pheA*^fbr^-*ppsA*-*pck* and pCS-P_lpp0.6_-*dpgA*-lac-*pheA*^fbr^-*ppsA*-*pck*-*talB*-*tktA*. Subsequently, the P_lpp0.6_ promoter in plasmid pCS-P_lpp0.6_-*dpgA*-lac-*pheA*^fbr^-*ppsA*-*pck*-*talB*-*tktA* was replaced by the P_lpp0.2_, P_lac_, P_lpp1.0_, and P_lpp4.0_ promoters respectively by homologous recombination, generating plasmids pCS-P_lpp0.2_-*dpgA*-lac-*pheA*^fbr^-*ppsA*-*pck*-*talB*-*tktA*, pCS-lac-*dpgA*-lac-*pheA*^fbr^-*ppsA*-*pck*-*talB*-*tktA*, pCS-P_lpp1.0_-*dpgA*-lac-*pheA*^fbr^-*ppsA*-*pck*-*talB*-*tktA*, and pCS-P_lpp4.0_-*dpgA*-lac-*pheA*^fbr^-*ppsA*-*pck*-*talB*-*tktA*. In addition, the *maeB*, *zwf*, *pntA*, *pntB*, and *nadK* genes were amplified from *E. coli* and cloned into pSA74 using the restriction sites *Kpn*I and *Bam*HI, yielding plasmids pSA-*maeB*, pSA-*zwf*, pSA-*pntAB*, and pSA-*nadK*, respectively. The *ppnK* gene was amplified from the genomic DNA of *Corynebacterium glutamicum* and similarly cloned into pSA74 via *Kpn*I and *Bam*HI, generating plasmid pSA-*ppnK*. The amino acid sequence of HmaS, SMDH and D-PhgAT are shown in Table S1.

### 2.4 Feeding experiments and *de novo* biosynthesis of D-PHG

In the substrate-feeding fermentation experiments, *E. coli* BW25113 and XYJ01 carrying plasmid pET-lac were first cultivated in 5 mL of LB medium. Then, 1 mL of each culture was inoculated into 50 mL M9Y medium and cultivated at 37°C with shaking at 220 rpm. When the OD_600_ reached 0.6, the cultures were shifted to 30°C, and PGA was then added at 2 g/L. Samples were collected every 12 h for OD_600_ measurement using a UV spectrophotometer, and metabolite contents were measured by HPLC. For *de novo* biosynthesis of D-PHG, the production strains were first cultivated in LB medium, after which 1 mL of the seed culture was inoculated into 50 mL M9Y medium. When the OD_600_ reached 2.0, IPTG was supplied at 0.5 mM, and the cultures were shifted to 30°C and incubated at 220 rpm. HPLC analysis was performed on samples taken every 12 h.

### 2.5 Genome modifications

Gene knockout and integration were performed using the CRISPR/Cas9 genome editing system [16]. The deleted genes included *pykA*, *pykF*, *tyrA*, *tyrR*, *tyrB*, *aspC*, and *ilvE*, while the integrated genes included *aroG*^fbr^, *aroB*, and *aroD* at the *ilvG* locus, *gdhA* at the *yeeL* locus, *nadK* at the *yciQ* locus, and *pntAB* at the *ylbE* locus. Briefly, the pCas9 plasmid was first transformed into the host strain, and the RED recombination system was induced by IPTG. The pTarget plasmid together with the homologous recombination fragment containing the target gene was then transformed into the resulting strain, followed by verification of positive clones. Subsequently, the pTarget plasmid was eliminated by adding 0.2% arabinose. The final strain was then cultivated overnight at 42°C to eliminate the pCas9 plasmid.

### 2.6 Fed-batch production of D-PHG

The fermentation strain was first cultivated in test tubes for 15 h and then transferred into 50 mL of M9Y seed medium, followed by incubation at 37°C in a shaking incubator for 8 h. The resulting seed culture was then used to inoculate 950 mL of fermentation medium in a 3-L bioreactor, composed of 3 g/L aspartate, 0.2 g/L tyrosine, 0.1 g/L each of leucine, isoleucine, phenylalanine, and valine, 10 g/L yeast extract, 15 g/L glucose, 2 g/L (NH_4_)_2_SO_4_, 3 g/L KH_2_PO_4_, 6.78 g/L Na_2_HPO_4_, and 2 mL of trace element solution (TES). The composition of TES was prepared according to a previous report [17]. The fermentation medium initially contained 15 g/L glucose. After the initial glucose was depleted, an 800 g/L glucose solution was fed to maintain the residual glucose concentration below 5 g/L. When the OD_600_ reached 10, IPTG was added at a final concentration of 0.5 mM, and the cultivation temperature was then shifted to 30°C. During fermentation, the pH was controlled at 6.9 and the dissolved oxygen (DO) level was maintained at 30%.

### 2.7 Fluorescence measurement

A single colony was grown overnight in LB medium. Subsequently, the resulting culture was then distributed into a 96-well microplate (Corning, NY, USA) at 10 μL per well, and each well contained 200 μL of M9Y medium, with antibiotics supplemented as required. The microplate was then incubated in a Synergy™ microplate reader (BioTek Instruments, VT, USA) at 30°C for 48 h. During cultivation, OD_600_ and fluorescence intensity were measured every 10 min, using excitation and emission wavelengths of 579 and 616 nm, respectively. All experiments were carried out in triplicate.

### 2.8 SDS-PAGE analysis

For protein solubility analysis, a single colony was grown overnight in 4 mL LB at 37°C and 200 rpm. Thereafter, the seed culture was inoculated into 100 mL LB medium at 1% (v/v) and cultivated to an OD_600_ of 0.6. Protein expression was then induced with 0.5 mM IPTG and the cells were cultured at 30°C for 12 h. The cells were then collected by centrifugation at 4°C (6000 rpm, 10 min), resuspended in lysis buffer and disrupted using a biological sample homogenizer. The crude extracts were subjected to another centrifugation step under the same conditions (6000 rpm, 10 min, 4°C). The resulting supernatant represented the soluble protein fraction, whereas the pellet was resuspended in lysis buffer as the insoluble fraction. Protein solubility was subsequently evaluated by SDS-PAGE analysis of both fractions.

### 2.9 Metabolites analysis

Chemical standards of D-PHG (99.5%), PGA (>95.0%), and S-MA (99.0%) were purchased from Shanghai Macklin Biochemical Co., Ltd. (Shanghai, China). External calibration curves were generated using working standards prepared by serial dilution to final concentrations of 0.05, 0.1, 0.5, 1.0 and 2.0 g/L. The concentrations of D-PHG, PGA, and S-MA in the fermentation broth were qualitatively and quantitatively analyzed by a SHIMADZU HPLC system equipped with a reversed-phase NanoChrom C18 column. Before HPLC analysis, fermentation samples were centrifuged at 13,000 rpm for 10 min to remove cells, and the resulting supernatants were filtered through a 0.22 μm membrane before HPLC analysis. The mobile phase consisted of methanol as solvent B and water containing 0.2% trifluoroacetic acid as solvent A. The column was operated at 40°C, and elution was performed using the following gradient program: 20% methanol for 0–7 min, 50% methanol for 7–15 min, 100% methanol for 15–17 min, and 20% methanol for 17–22 min. The flow rate was maintained at 1.0 mL/min, and the injection volume for each sample was 10 μL. Detection was performed at dual wavelengths of 250 and 260 nm, and calibration curves were generated from peak areas at 260 nm for metabolite quantification. All reported metabolite titers represent extracellular concentrations in the cell-free fermentation supernatant.

## 3 Results and discussion

### 3.1 Constructing an initial strain for D-PHG synthesis

**Fig. 2:**
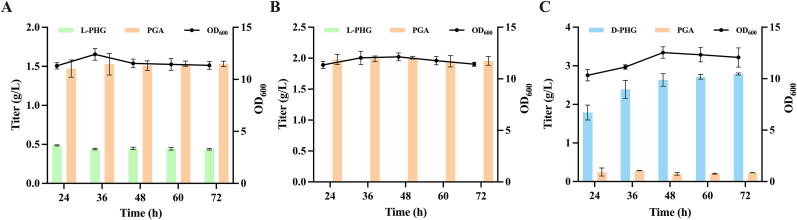
Constructing an initial D-PHG-producing strain. (A) The production of the by-product L-PHG by the strain XYJ06 (*E. coli* BW25113 harboring the empty plasmid pET-lac); (B) The production of the by-product L-PHG by the strain XYJ07 (generated by deleting the genes encoding endogenous aminotransferases AspC, IlvE, and TyrB in the strain XYJ06); (C) The production of the target product D-PHG by the strain XYJ08 (XYJ02 harboring plasmids pET-lac-*hmaS*-*hmaS*-*mdlB*-lac-*hmaS* and pCS-P_lpp0.6_-*dpgA*). All experiments were performed with three biological replicates. Data are presented as mean ± standard deviation.

Given that *E. coli* harbors multiple endogenous aminotransferases capable of converting α-keto acids into l-amino acids, we first investigated whether these endogenous enzymes would interfere with the specific biosynthesis of D-PHG. The empty vector pET-lac was introduced into *E. coli* BW25113 to generate strain XYJ06. When 2 g/L PGA was supplemented exogenously as the substrate, 0.44 g/L L-PHG was detected after 72 h of fermentation, with 1.53 g/L PGA remaining (Fig. 2A), indicating that endogenous aminotransferases in *E. coli* were indeed able to convert the precursor PGA into the by-product L-PHG. Because PGA is structurally similar to PPA, the precursor of l-phenylalanine, endogenous aminotransferases involved in aromatic amino acid metabolism may accept PGA as a non-native substrate. Therefore, *aspC*, *tyrB*, and *ilvE* were selected as the main knockout targets to eliminate the competing transamination reaction from PGA to L-PHG. To verify this hypothesis and eliminate L-PHG formation, the endogenous α-aminotransferase genes *aspC*, *ilvE*, and *tyrB* were deleted from *E. coli* BW25113, yielding strain XYJ01. The empty vector pET-lac was subsequently introduced into strain XYJ01 to obtain strain XYJ07. After supplementation with 2 g/L PGA and fermentation for 72 h, no L-PHG formation was detected and no PGA consumption was observed (Fig. 2B), demonstrating that deletion of the endogenous aminotransferase genes *tyrB*, *ilvE*, and *aspC*, effectively abolished the side reaction leading to L-PHG formation from PGA. Therefore, strain XYJ01 was selected for subsequent D-PHG biosynthesis.

An adequate precursor supply is essential for efficient D-PHG biosynthesis. Thus, the pyruvate kinase genes *pykA* and *pykF* were deleted in strain XYJ01 to reduce PEP consumption toward pyruvate [18], thereby increasing intracellular PEP availability. In addition, the prephenate dehydrogenase gene *tyrA* was deleted to block the conversion of prephenate (PRE) to 4-hydroxyphenylpyruvate (HPPA) [19], and the bifunctional transcriptional regulator gene *tyrR* was removed to relieve feedback repression on the key shikimate pathway genes such as *aroG* and *aroL* [20]. To further enhance carbon flux toward the shikimate pathway, *aroG*^fbr^ (D146N) gene encoding 3-deoxy-D-arabino-heptulosonate-7-phosphate synthase, together with *aroB* encoding 3-dehydroquinate synthase and *aroD* encoding 3-dehydroquinate dehydratase [21], were genomically integrated into the *ilvG* site in strain XYJ01, generating strain XYJ02. In our previous study, pseudogene sites such as *ilvG*, *yeeL*, *yciQ*, and *ylbE* were systematically evaluated and identified as suitable chromosomal integration sites for stable and efficient gene expression in *E. coli* [22]. These loci supported reliable expression of integrated genes without affecting cell growth and were therefore selected for introducing different pathway enzymes in this study. Previous studies have shown that tandem expression of three copies of *hmaS* from *A. orientalis* on a high-copy-number plasmid could markedly enhance the conversion of PPA to S-MA [23]. The SMDH enzyme from *P. putida* catalyzes the conversion of S-MA to PGA depending on FMN, thereby avoiding the formation of hydrogen peroxide [24]. In addition, D-PhgAT from *P. stutzeri* ST-201 can efficiently catalyze the conversion of PGA to D-PHG, with a conversion rate as high as 93% [25]. On this basis, the plasmids pET-lac-*hmaS*-*hmaS*-*mdlB*-lac-*hmaS* and pCS-P_lpp0.6_-*dpgA* were constructed for D-PHG biosynthesis. Co-transformation of pET-lac-*hmaS*-*hmaS*-*mdlB*-lac-*hmaS* and pCS-P_lpp0.6_-*dpgA* into strain XYJ02 generated strain XYJ08. After 72 h of fermentation, strain XYJ08 produced 2.79 g/L D-PHG (Fig. 2C). We also observed minimal accumulation of the precursor PGA (0.23 g/L), suggesting that insufficient precursor supply and the relatively low catalytic efficiency of D-PhgAT constitute key bottlenecks in D-PHG production.

### 3.2 Redirecting carbon flux toward D-PHG over-production

**Fig. 3:**
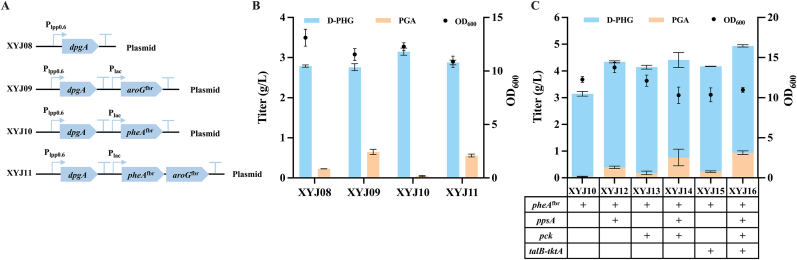
Redirecting carbon metabolic flux for D-PHG over-production. (A) Schematic diagram of plasmid construction in production strains XYJ08-11; (B) Strengthening D-PHG biosynthesis by relieving feedback inhibition in the shikimate pathway (XYJ08 harboring pCS-P_lpp0.6_-*dpgA*; XYJ09 harboring pCS-P_lpp0.6_-*dpgA*-lac-*aroG*^fbr^; XYJ10 harboring pCS-P_lpp0.6_-*dpgA*-lac-*pheA*^fbr^; XYJ11 harboring pCS-P_lpp0.6_-*dpgA*-lac-*pheA*^fbr^-*aroG*^fbr^); (C) Promoting D-PHG biosynthesis by increasing intracellular supplies of the precursors PEP and E4P (XYJ12 harboring pCS-P_lpp0.6_-*dpgA*-lac-*pheA*^fbr^-*ppsA*; XYJ13 harboring pCS-P_lpp0.6_-*dpgA*-lac-*pheA*^fbr^-*pck*; XYJ14 harboring pCS-P_lpp0.6_-*dpgA*-lac-*pheA*^fbr^-*ppsA*-*pck*; XYJ15 harboring pCS-P_lpp0.6_-*dpgA*-lac-*pheA*^fbr^-*talB*-*tktA*; XYJ16 harboring pCS-P_lpp0.6_-*dpgA*-lac-*pheA*^fbr^-*ppsA*-*pck*-*talB*-*tktA*). All experiments were performed with three biological replicates. Data are presented as mean ± standard deviation.

We first attempted over-expression of the key genes in the shikimate pathway to improve precursor supply. The feedback inhibition resistant *aroG*^fbr^ and *pheA*^fbr^ were individually overexpressed in strain XYJ08, generating strains XYJ09 and XYJ10 (Fig. 3A), which produced 2.76 g/L and 3.15 g/L D-PHG, respectively (Fig. 3B). Co-overexpression of *aroG*^fbr^ and *pheA*^fbr^ in strain XYJ08 yielded strain XYJ11 (Fig. 3A), in which the D-PHG titer decreased to 2.88 g/L (Fig. 3B). Therefore, strain XYJ10 was selected as the parental strain for subsequent engineering.

Then, we engineered PEP supply to improve D-PHG synthesis. In central carbon metabolism, phosphoenolpyruvate synthase PpsA can convert pyruvate back to PEP [26]. The *ppsA* gene was introduced into plasmid pCS-P_lpp0.6_-*dpgA*-lac-*pheA*^fbr^ to generate plasmid pCS-P_lpp0.6_-*dpgA*-lac-*pheA*^fbr^-*ppsA*, which was then co-transformed with pET-lac-*hmaS*-*hmaS*-*mdlB*-lac-*hmaS* into strain XYJ02 to generate strain XYJ12. The D-PHG titer enhanced to 4.34 g/L (Fig. 3C). In addition, phosphoenolpyruvate carboxykinase Pck can regenerate PEP from oxaloacetate [27]. The *pck* gene was cloned into plasmid pCS-P_lpp0.6_-*dpgA*-lac-*pheA*^fbr^ to generate pCS-P_lpp0.6_-*dpgA*-lac-*pheA*^fbr^-*pck*. Co-transformation of this plasmid with pET-lac-*hmaS*-*hmaS*-*mdlB*-lac-*hmaS* into strain XYJ02 yielded strain XYJ13, which produced 4.17 g/L D-PHG (Fig. 3C). Finally, the genes *ppsA* and *pck* were co-expressed to construct plasmid pCS-P_lpp0.6_-*dpgA*-lac-*pheA*^fbr^-*ppsA*-*pck*, which was co-transformed with pET-lac-*hmaS*-*hmaS*-*mdlB*-lac-*hmaS* into strain XYJ02 to obtain strain XYJ14. As a result, the D-PHG titer of strain XYJ14 was further increased to 4.41 g/L, 40% higher than that of the control strain XYJ10 (Fig. 3C).

Next, we engineered E4P supply to further improve D-PHG production. In the pentose phosphate pathway, transketolase TktA and transaldolase TalB participate in E4P formation [13]. Therefore, the *talB*-*tktA* expression cassette was cloned into plasmid pCS-P_lpp0.6_-*dpgA*-lac-*pheA*^fbr^ to generate plasmid pCS-P_lpp0.6_-*dpgA*-lac-*pheA*^fbr^-*talB*-*tktA*, which was co-transformed with pET-lac-*hmaS*-*hmaS*-*mdlB*-lac-*hmaS* into strain XYJ02 to obtain strain XYJ15. This strain produced 4.14 g/L D-PHG (Fig. 3C). Finally, the genes *ppsA*, *pck* and *talB*-*tktA* were co-expressed to construct plasmid pCS-P_lpp0.6_-*dpgA*-lac-*pheA*^fbr^-*ppsA*-*pck*-*talB*-*tktA*, which was co-transformed with pET-lac-*hmaS*-*hmaS*-*mdlB*-lac-*hmaS* into strain XYJ02 to generate strain XYJ16. This strain exhibited the highest D-PHG titer, reaching 4.94 g/L (Fig. 3C), demonstrating a 56.8% enhancement compared with the control strain XYJ10. However, 0.94 g/L PGA was still detected in the culture medium, suggesting that the conversion of PGA to D-PHG remained the key bottleneck for D-PHG production.

### 3.3 Strengthening the conversion of PGA to D-PHG to promote D-PHG biosynthesis

**Fig. 4:**
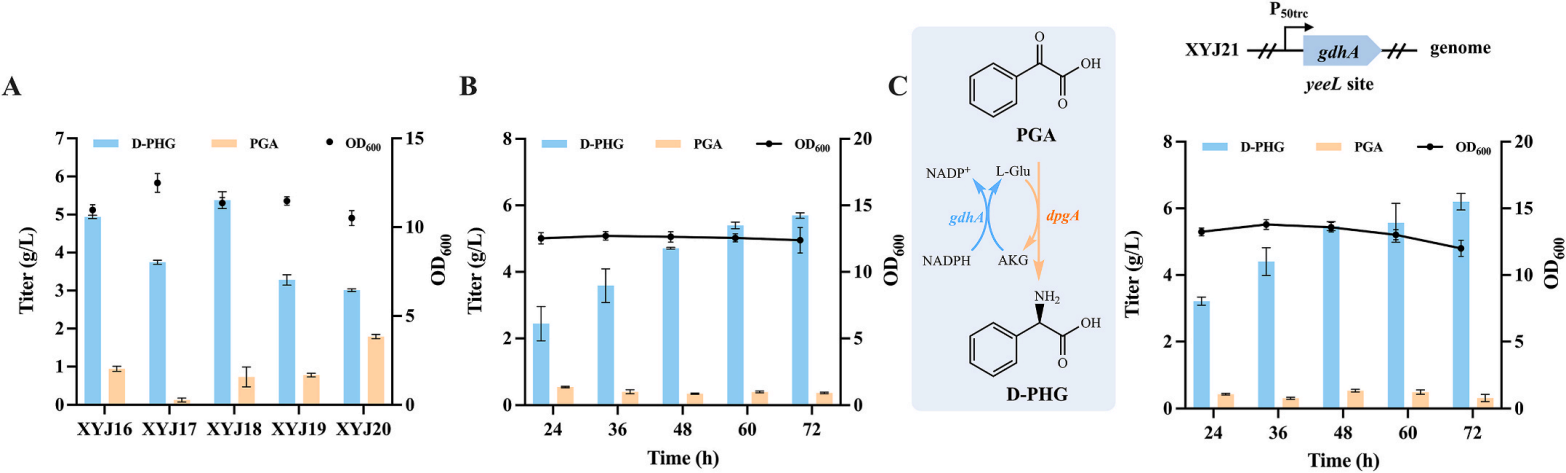
Enhanced D-PHG production by strengthening aminotransferase expression and amino group donor supply. (A) Optimization of D-PhgAT expression using four promoters with different strengths (XYJ16 carrying promoter P_lpp0.6_; XYJ17 carrying promoter P_lpp0.2_; XYJ18 carrying promoter P_lac_; XYJ19 carrying promoter P_lpp1.0_; XYJ20 carrying promoter P_lpp4.0_); (B) D-PHG production by strain XYJ18 after exogenous addition of 3 g/L glutamine; (C) D-PHG production by strain XYJ21 after increasing intracellular amino group donor supply through genomic integration of *gdhA*. All experiments were performed with three biological replicates. Data are presented as mean ± standard deviation.

To address the potential metabolic limitations in the transamination reaction from PGA to D-PHG, we focused on optimizing two key factors affecting this step, the expression level of D-PhgAT and the effective supply of the amino group donor. First, the promoter strength driving D-PhgAT expression was optimized. The original P_lpp0.6_ promoter was replaced with four promoters with different strengths, including P_lpp0.2_ < P_lac_ < P_lpp1.0_ < P_lpp4.0_ (Fig. S1), generating strains XYJ17-XYJ20, respectively. As shown in Fig. 4A, after 72 h of shake-flask fermentation, strain XYJ17, in which D-PhgAT was driven by the P_lpp0.2_ promoter, produced 3.75 g/L D-PHG, which was below the level observed in the control strain XYJ16 (P_lpp0.6_). This decrease is likely due to insufficient D-PhgAT expression under the relatively weak promoter. In contrast, strain XYJ18, harboring D-PhgAT under control of the P_lac_ promoter, exhibited the highest D-PHG production, reaching 5.38 g/L. Surprisingly, strains XYJ19 and XYJ20, in which D-PhgAT was driven by the stronger promoters P_lpp1.0_ and P_lpp4.0_, showed reduced D-PHG titers of 3.28 g/L and 3.01 g/L, respectively (Fig. 4A). SDS-PAGE analysis indicated that expression under the stronger promoters led to increased formation of inclusion bodies (Fig. S2), suggesting that excessive expression may overwhelm the cellular folding machinery, thereby impairing enzyme activity and limiting product formation. Furthermore, even in strain XYJ18, substantial accumulation of the precursor PGA (0.73 g/L) was observed (Fig. 4A), indicating that the conversion of PGA to D-PHG remains a rate-limiting step.

Next, we sought to enhance amino group donor availability to further improve the conversion of PGA to D-PHG. Previous studies have shown that D-PhgAT utilizes l-glutamate (L-Glu) as the amino group donor [25]. As an initial strategy, exogenous L-Gln was supplemented to increase amino donor group supply. During fermentation of strain XYJ18, the addition of 3 g/L L-Gln resulted in an increased D-PHG titer of 5.69 g/L after 72 h, accompanied by a reduction in PGA accumulation to 0.37 g/L (Fig. 4B). These results indicate that enhancing amino donor group availability can partially alleviate the limitation of this conversion step. Building on this, we further strengthened endogenous L-Glu biosynthesis through metabolic engineering. Glutamate dehydrogenase GDH from *E. coli* MG1655 catalyzes the reductive amination of α-ketoglutarate (AKG) with free NH_4_^+^ to form L-Glu using NADPH as a cofactor [28]. Accordingly, the gene *gdhA* was integrated into the *yeeL* locus of strain XYJ02, generating strain XYJ03. Subsequently, plasmids pET-lac-*hmaS*-*hmaS*-*mdlB*-lac-*hmaS* and pCS-lac-*dpgA*-lac-*pheA*^fbr^-*ppsA*-*talB*-*tktA*-*pck* were co-transformed into strain XYJ03, yielding strain XYJ21. After 72 h of fermentation, strain XYJ21 produced 6.20 g/L D-PHG, with 0.32 g/L PGA still accumulating (Fig. 4C). This result suggests that, although L-Glu supply was improved, amino donor group availability remains a limiting factor and requires further optimization.

### 3.4 Enhancing intracellular NADPH supply for improved D-PHG biosynthesis

In the *de novo* D-PHG biosynthetic pathway, intracellular NADPH supply plays a crucial role in regulating overall metabolic flux. The GDH catalyzed conversion of AKG to L-Glu requires NADPH (Fig. 4C). In addition, the conversion of 3-dehydroshikimate to shikimate in the shikimate pathway also depends on NADPH as a cofactor [29]. We therefore hypothesized that enhancing intracellular NADPH availability would promote D-PHG biosynthesis by simultaneously strengthening precursor formation and amino group donor availability.

**Fig. 5:**
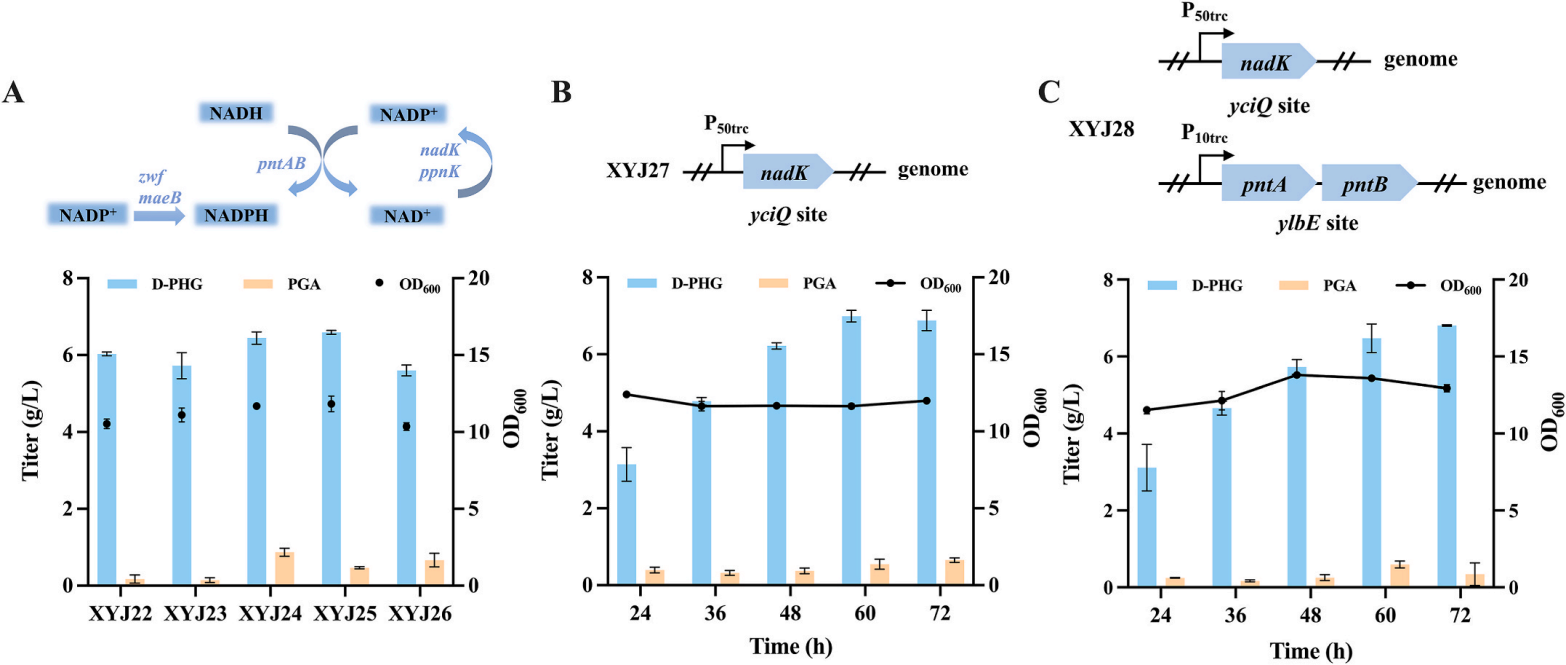
Strengthening D-PHG biosynthesis by enhancing intracellular NADPH supply. (A) Improving D-PHG production by overexpression of the genes involved in NADPH generation (XYJ22 harboring pSA-lac-*maeB*; XYJ23 harboring pSA-lac-*zwf*; XYJ24 harboring pSA-lac-*pntAB*; XYJ25 harboring pSA-lac-*nadK*; XYJ26 harboring pSA-lac-*ppnK*); (B) D-PHG production by strain XYJ27 with genomic integration of *nadK*; (C) D-PHG production by strain XYJ28 with further genomic integration of *pntAB*. All experiments were performed with three biological replicates. Data are presented as mean ± standard deviation.

To test this hypothesis, we implemented multiple strategies to enhance NADPH regeneration. The NADP^+^-dependent malate dehydrogenase MaeB directly generates NADPH through malate decarboxylation [30], whereas glucose-6-phosphate dehydrogenase Zwf produces NADPH by converting glucose-6-phophate to 6-phosphogluconolactone in the pentose phosphate pathway [31]. Individual overexpression of *maeB* and *zwf* in strain XYJ21 generated strains XYJ22 and XYJ23, respectively, both of which showed reduced D-PHG titers of 6.03 g/L and 5.72 g/L (Fig. 5A). The gene *pntAB* from *E. coli*, encoding a membrane-bound transhydrogenase, converts NADH into NADPH using the proton motive force [32]. Overexpression of *pntAB* in the strain XYJ21 generated strain XYJ24, in which the D-PHG titer increased to 6.44 g/L, with 0.87 g/L of PGA accumulated (Fig. 5A). The genes *nadK* from *E. coli* [32] and *ppnK* from *Corynebacterium glutamicum* [33], both encoding NAD kinases, convert NAD^+^ into NADP^+^, thereby increasing the intracellular NADP(H) pool and supporting NADPH synthesis. Overexpression of *nadK* and *ppnK* in XYJ21 yielded strains XYJ25 and XYJ26, respectively. The results demonstrated that the strain XYJ25 harboring *nadK* exhibited the best performance, producing 6.59 g/L of D-PHG with 0.47 g/L of PGA accumulation (Fig. 5A).

Given that both *nadK* and *pntAB* improved D-PHG production, they were subsequently integrated into the chromosome to reduce plasmid burden and metabolic load. Integration of *nadK* into the *yciQ* locus of strain XYJ03 generated strain XYJ04. After introduction of plasmids pET-lac-*hmaS*-*hmaS*-*mdlB*-lac-*hmaS* and pCS-lac-*dpgA*-lac-*pheA*^fbr^-*ppsA*-*talB*-*tktA*-*pck*, strain XYJ27 produced 6.98 g/L of D-PHG with 0.54 g/L of PGA accumulation (Fig. 5B), representing the highest titer achieved. To further strengthen NADPH regeneration, *pntAB* was integrated into the *ylbE* locus of XYJ04, generating XYJ05, which was subsequently transformed with the D-PHG production plasmids to obtain XYJ28. However, further integration of *pntAB* did not further improve D-PHG titer, and strain XYJ28 reached 6.81 g/L with 0.34 g/L of PGA accumulation (Fig. 5C).

### 3.5 Fed-batch fermentation for D-PHG production in a 3-L bioreactor

**Fig. 6:**
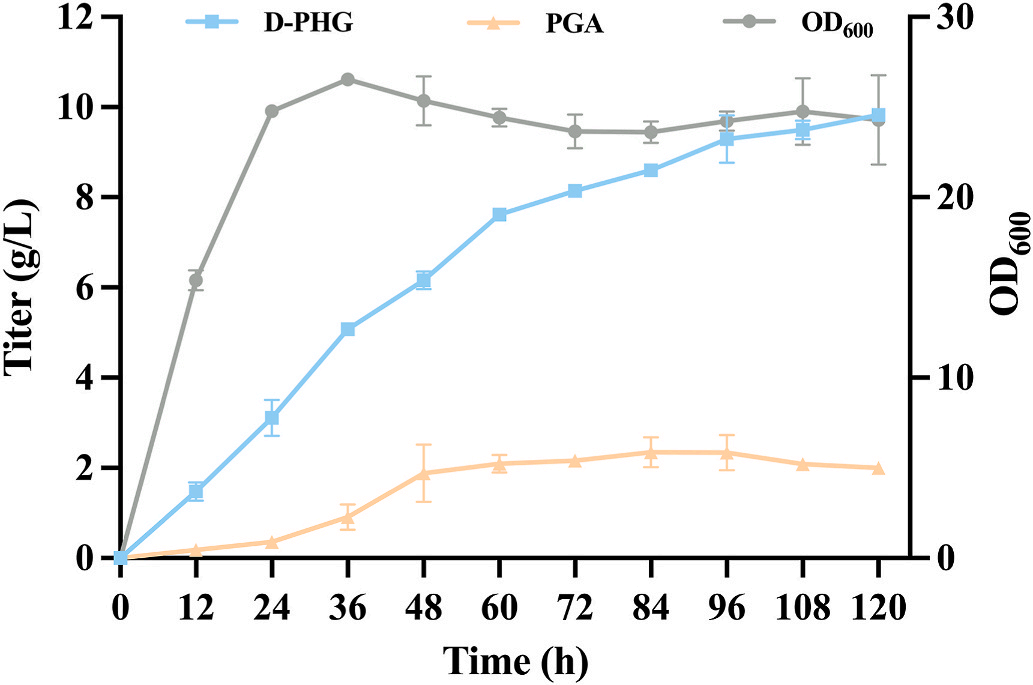
Fed-batch production of D-PHG using strain XYJ27 in a 3-L bioreactor. Blue squares represent D-PHG titer (g/L), orange triangles represent PGA titer (g/L), gray circles represent cell growth (OD_600_). All experiments were performed with two biological replicates. Data are presented as mean ± standard deviation.

To further examine the scalability of strain XYJ27, fed-batch fermentation was conducted in a 3-L bioreactor. The fermentation temperature was initially set at 37°C, with DO maintain at 30%, and 3 g/L Gln was supplemented as the amino group donor. Glucose was supplied at an initial concentration of 15 g/L. Once glucose was exhausted, an 800 g/L glucose feed was supplied to keep the residual glucose level below 5 g/L. When the OD_600_ reached 10, IPTG was introduced for protein induction after which the temperature was subsequently shifted to 30°C for continued cultivation. As shown in Fig. 6, the strain exhibited a rapid growth, with OD_600_ reaching a maximum of 26.5 at 36 h. D-PHG production increased continuously and finally reached 9.83 g/L at 120 h, representing the highest level reported so far. Notably, the precursor PGA accumulated during the entire fermentation process, reaching 2.01 g/L at the end of cultivation. This result indicates that the transamination step from PGA to D-PHG remains inefficient and constitutes a key bottleneck limiting further improvement in D-PHG production.

## 4 Conclusions

Microbial cell factories provide a sustainable and efficient platform for the green production of value-added chemicals. D-PHG is a valuable chiral intermediate widely used in the pharmaceutical industry. However, its microbial production has been constrained by low titers. In this work, we constructed a *de novo* pathway for D-PHG biosynthesis in *E. coli* and systematically enhanced its performance through a series of metabolic engineering strategies, including elimination of competing transamination pathways, reinforcement of precursor supply, optimization of key enzyme expression, enhancement of amino group donor availability, and improvement of intracellular NADPH regeneration. Through these efforts, D-PHG production was progressively increased, and subsequent fed-batch fermentation in a 3-L bioreactor achieved a final titer of 9.83 g/L, representing the highest level reported so far for *de novo* D-PHG biosynthesis. Overall, this study establishes robust and scalable microbial system for D-PHG biosynthesis and offers a generalizable framework for the synthesis of other non-natural aromatic amino acids and their derivatives.

Although this study significantly improved D-PHG production, further optimization is required for industrial implementation. First, the continuous accumulation of PGA during fed-batch fermentation indicates that the transamination step remains the primary bottleneck. Future efforts could focus on protein engineering of D-PhgAT to improve its catalytic performance. Second, the maximum cell density reached only an OD_600_ of 26.5, suggesting that cell growth remained constrained during scale-up fermentation. A possible explanation is that the deletion of *aspC*, *tyrB*, *ilvE*, and *tyrA*, which are involved in amino acid biosynthesis, may have impaired cellular growth under high cell density conditions. Consistent with this hypothesis, supplementation with amino acid mixtures partially improved cell growth, although it did not fully restore biomass accumulation. Further studies, including systematic nutrient supplementation and gene complementation experiments, will be required to verify the contribution of these gene deletions to the observed growth limitation. Alternatively, dynamic regulation strategies that allow normal expression of these genes during the growth phase and repress their expression during the production phase may provide an effective approach to balance biomass accumulation and D-PHG production.

## CRediT authorship contribution statement

**Yajuan Xiong:** Data curation, Formal analysis, Investigation, Writing – original draft. **Zhentong Shang:** Data curation, Investigation. **Liangyu Lu:** Data curation, Writing – review & editing. **Tong Wang:** Data curation, Writing – review & editing. **Guang Cai:** Data curation, Writing – review & editing. **Xiaolin Shen:** Writing – review & editing. **Xinxiao Sun:** Writing – review & editing. **Jia Wang:** Conceptualization, Formal analysis, Supervision, Writing – original draft. **Qipeng Yuan:** Supervision, Writing – review & editing.

## Declaration of competing interest

The authors declare that they have no known competing financial interests or personal relationships that could have appeared to influence the work reported in this paper.
