# Supplementary material for: Metabolic engineering of Escherichia coli for efficient production of d-phenylglycine
Source: Synth Syst Biotechnol. 2026 Aug 7;17:106–14. doi: 10.1016/j.synbio.2026.07.010 (PMC13475659; doi:10.1016/j.synbio.2026.07.010)
Supplement: Supplementary file 1 [file mmc1.docx]

**Metabolic Engineering of *Escherichia coli* for Efficient Production of D-phenylglycine**

Yajuan Xiong^a^, Zhentong Shang^a^, Liangyu Lu^b^, Tong Wang^a^, Guang Cai^b^, Xiaolin Shen^a^, Xinxiao Sun^a^, Jia Wang^a,c,*^ and Qipeng Yuan^a^

**Supplementary Information**

**Figure S1.** Comparison of promoter strengths of P_lpp0.2_, P_lpp0.6_, P_lac_, P_lpp1.0_ and P_lpp4.0_. Fluorescence intensity of *mCherry* was used as a reporter to evaluate the promoter activities in *E. coli* BW25113 strains carrying pCS-P_lpp0.2_-*mCherry*, pCS-P_lpp0.6_-*mCherry*, or pCS-P_lac_-*mCherry*, pCS-P_lpp1.0_-*mCherry*, pCS-P_lpp4.0_-*mCherry*, respectively. Data are presented as mean ± standard deviation.

**Figure S2.** SDS-PAGE analysis of D-PhgAT expression level in XYJ02-derived recombinant strains harboring plasmids pCS27, pCS-P_lpp0.6_-*dpgA*, pCS-P_lpp0.2_-*dpgA*, pCS-P_lac_-*dpgA*, pCS-P_lpp1.0_-*dpgA* and pCS-P_lpp4.0_-*dpgA*, respectively. S represents the soluble protein, and I represent the insoluble protein. The predicted molecular weight of D-PhgAT is approximately 49.0 kDa. The red outline indicates the position of the target protein band.

**Table S1.** **The amino acid sequence of HmaS, SMDH and D-PhgAT.**

| **Enzymes** | **Source** | **Sequence (5’-3’)** |
| --- | --- | --- |
| HmaS | *Amycolatopsis orientalis* | MQNFEIDYVEMYVANLEAAASGWMDKYGFSVTATDRSADHRSVTLRHSAIALVLTEPLSDRHPGATYLQTHGDGVADIALRTPDVAAAFEAAVKAGAEPVREPEKRADSVVTATVRGFGDVVHTLIQSDAAEETPRGKGGVELGVIDHFAVCLNAGDLGPTVAFYERALGFKQIFEEHIVVGAQAMNSTVVQSTSGAVTLTLIEPDKTADPGQIDDFIKEHHGSGVQHIAFTSPDAVRAVKELSARGVEFLKTPDAYYDLLGERIELETHSLDDLRETKLLADEDHGGQLFQIFTASTHPRKTIFFEIIERQGAGTFGSSNIKALYEAVELERTGQSKLGPVRR* |
| SMDH | *Pseudomonas putida* | MSRNLFNVEDYRKLAQKRLPKMVYDYLEGGAEDEYGVKHNRDVFQQWRFKPKRLVDVSRRSLQAEVLGKRQSMPLLIGPTGLNGALWPKGDLALAQAATKAGIPFVLSTASNMSIEDLARQCDGDLWFQLYVIHREIAQGMVLKALHSGYTTLVLTTDVAVNGYRERDLHNRFKMPMSYTPKVMLDGCLHPRWSLDLVRHGMPQLANFVSSQTSSLEMQAALMSRQMDASFNWEALRWLRDLWPHKLLVKGLLSAEDADRCIAEGADGVILSNHGGRQLDCAVSPMEVLAQSVAKTGKPVLIDSGFRRGSDIVKALALGAEAVLLGRATLYGLAARGETGVGEVLTLLKADIDRTLAQIGCPDITSLSPDYLQSEGVTNTAPVDHLIGKGTHA* |
| D-PhgAT | *P. stutzeri* ST-201 | MSILNDYKRKTEGSVFWAQRARSVMPDGVTADTRVFDPHGLFISDAQGVHKTDVDGNVYLDFFGGHGALVLGHGHPRVNAAIAEALSHGVQYAASHPLEVRWAERIVAAFPSIRKLRFTGSGTETTLLALRVARAFTGRRMILRIATHYHGWHDFSASGYNSHFDGQPAPGVLPEIAKNTLLIRPDDIEGMREVFAQHGSDIAAFIAEPVGSHFGVTPVSDSFLREGAELARQYGALFILDEVISGFRVGNHGMQALLDVQPDLTCLAKASAGGLPGGILGGREDVMGVLSRGSDRKVLHQGTFTGNPITAAAAIAAIDTILEDDVCAKINDLGQFAREAMNHLFARKGLNWLAYGRFSGFHLMPGLPPNTTDTGSITRAEVARPDVKMIAAMRMALILEGVDIGGRGSVFLSAQHEREHVEHLVTTFDRVLDRLADENLLSWQPTNLSGNQS* |
